# Supplementary material for: Joint Neuropsychological Assessment through Coma/Near Coma and Level of Cognitive Functioning Assessment Scales Reduces Negative Findings in Pediatric Disorders of Consciousness
Source: Brain Sci. 2020 Mar 12;10(3):162. doi: 10.3390/brainsci10030162 (PMC7140001; doi:10.3390/brainsci10030162)
Supplement: Supplementary file 1 [file brainsci-10-00162-s001.pdf]

Supplementary material

Extended Methods

2.1. Participants

Participants were recruited from a cohort of children and adolescents with acquired brain injury (ABI) referred to Scientific Institute «Eugenio Medea» for a clinical and functional assessment and for rehabilitation in the period between 2008 and 2016.

Inclusion criteria were: (i) age at first assessment between 0 and 18 years; (ii) time between injury and first assessment <3 months; (iii) documented evidence of a severe ABI with DOC of traumatic, anoxic, vascular or infectious etiology, as confirmed by a Glasgow Coma Scale (GCS) [1] score ≤8 at insult; (iv) absence of congenital pathology or disability previous to the injury; (v) medical records sufficiently detailed to determine the injury severity and neurological findings.

During the study period, 147 pediatric patients with a diagnosis of UWS/VS or MCS were admitted to our center. Diagnosis was performed by the clinical team, on the basis of the Aspen Neurobehavioral Conference Workgroup established definitions of DOC [2], evoked potentials, and radiological information. Of note, *Rappaport Coma/Near Coma Scale* (CNCS) [3] and *Level of Cognitive Functioning Assessment Scale* (LOCFAS) [4,5] were not used for the diagnosis.

Of the selected patients, 92 met the inclusion criteria and made up the final study sample. The remaining 55 patients were excluded for the following reasons:

35 patients received the first assessment later than 3 months past injury;

6 patients were older than 18 years at the time of the assessment;

7 patients presented with incompatible diagnosis (e.g., brain tumor);

7 patients presented with a congenital pathology.

2.2. Measures and Procedures

2.2.1. Measures description

Coma/Near Coma Scale (CNCS) was developed in order to assess small clinical changes in patients with severe brain injuries who function at very low levels characteristics of near-VS and VS (now UWS). It consists of 11 items assessing responsivity to auditory, visual, olfactory, tactile, and painful stimuli as well as command following and vocalization. Each item is rated with a score of 0, 2, or 4, depending on quality and/or consistency of response. Lower scores indicate higher level of responsiveness. Overall summation of the scores at the 11 items is defined 'Total CNCS Score', which ranges from 0 to 44; the 11 item scores are further averaged to obtain the 'Average CNCS Score', which thus spans from 0 to 4; the 'Average CNCS Score' can be rounded and assigned to a 'CNCS Level', which also spans integers from 0 to 4. The CNCS was designed to provide reliable and valid assessment of the evolution of the responsivity of patients with severe brain lesions. Finally, previous studies have reported the administration of this instrument to children [7–9].

Administration of Level of Cognitive Functioning Assessment Scale (LOCFAS) [5] relies on the behavioral observation of the patients' interactions with the environment, either in the absence or presence of changing stimuli. This scale provides a detailed description of a patient's cognitive-behavioral functioning during recovery and a measure of the patient's progress in the early post-acute stage. It is made up of 9 levels, each of them composed by different domains: attention for environment, responses to stimuli, behavior, information processing, execution of commands, awareness of self, time orientation, ability to perform self-care activities, ability to converse, ability to learn new information. The first 5 levels are considered as measure of the cognitive functioning in patients with disorder of consciousness: level 1 ('Non responsiveness'), level 2 ('Generalized answers'), level 3 ('Localized answers'), level 4 ('Confused-agitated') and level 5 ('Inappropriate, not agitated').

The improvement in cognitive functioning results from both the progression across LOCFAS levels and the increase in the saturation percentage (number of domains satisfied for certain level / total number of domains for this level).

2.2.2. Procedures

After admission to our center, the primary researcher (S.S.) contacted each consenting caregiver to arrange an assessment at the hospital. Demographic information was collected during a semi-structured interview. GCS recorded by the rescue team (or in the emergency room) was collected. GOS-E score was assessed by each patient's lead physician at admission to the rehabilitation center. Further medical details were obtained from medical records. CNCS and LOCFAS were delivered by two experienced neuropsychologists (K.C. and C.F.) on the same day (when delivered both).

All the patients were proposed a rehabilitation treatment according to the protocol used in our Intensive Rehabilitation Unit. Based on their clinical condition and at least five days a week, they received:

- two daily sessions of physical therapy lasting 45 minutes each. Physical rehabilitation aims at preventing secondary damage such as muscle retractions and joint deformities, enhancing normalization of muscular tone, correcting posture and alignment;
- one daily session of oro-facial therapy lasting 45 minutes. Oro-facial therapy aims at restoring normal feeding;
- two to three daily cognitive-behavioral sessions of 10-20 minutes each, as described in detail in previous studies by our group [10,11]. Since the early days of recovery, cognitive-behavioral stimulations may help patients in a UWS/VS and MCS to reinforce their adaptive responses - either spontaneous or elicited by multisensory stimulations - and rebuild their behavioral repertoire. The underlying principle is *conditioning*, and techniques can be divided into two large categories: techniques positively reinforcing all the spontaneous adaptive responses and techniques favoring the acquisition and generalization of new behavioral patterns. Cognitive-behavioral stimulations also include techniques and procedures to reduce inappropriate behaviors. Individualized goals are specifically related to the assessed level of functioning.
- one group session of cognitive-behavioral stimulations daily.

Furthermore, once a week caregivers took part in a psychoeducational intervention aimed at involving the patients' families in psycho-stimulation.

Extended Results

Supplementary Table S1. Clinical and demographic characteristics of the sub-sample of patients having both CNCS and LOCFAS assessments, 3 and 6 months after injury. Table includes data divided by aetiology.

	**Total Sample (n=54)**		**Traumatic (TBI) (n=37)**		**Non Traumatic (NTBI) (n=17)**		**Statistics**
	**Mean**	**SD**	**Mean**	**SD**	**Mean**	**SD**	***p* (t-test)**
**Age at Injury (Months)**	130.1	53.1	146.2	51.8	95.2	37.6	**$<1 \times 10^{-3}$ ****
	**Median**	**Range**	**Median**	**Range**	**Median**	**Range**	***p* (Wilcoxon test)**
**GCS Score**	4	2-8	4	2-7	4	3-8	0.644
**GOS-E Score**	2	2-3	2	2-3	2	2-2	0.163
	n	**%**	***n***	**%**	***n***	**%**	***p* (2)**
**Gender**							
**Male**	35	64.8	23	62.2	12	70.6	0.760
**Female**	19	35.2	14	37.8	5	29.4	
**Need of neurosurgery**	32	59.3	26	70.3	6	35.3	**0.035***
**Tracheotomy**	32	59.3	24	64.9	8	47.1	0.368
**Feeding Disorders**							
**Absence of disorders**	0	0.0	0	0.0	0	0.0	0.115
**Dysphagia**	2	3.7	2	5.4	0	0.0	
**Naso Gastric Tube (NGT)**	21	38.9	11	29.7	10	58.8	
**Percutaneous endoscopic gastrostomy (PEG)**	31	57.4	24	64.9	7	41.2	
**Paroxysmal Sympathetic Hyperactivity Episodes**	24	44.4	15	40.5	9	52.9	0.372
**Motor Impairment**							
**Absence of impairment**	1	1.9	1	2.7	0	0.0	0.382
**Motor retardation**	1	1.9	1	2.7	0	0.0	
**Quadriparesis**	51	94.3	35	94.6	16	94.1	
**Ataxia**	1	1.9	0	0.0	1	5.9	
**Previous Rehabilitation**	1	1.9	0	0.0	0	0.0	0.621

** highly significant at *p*-value < 0.001.

3.2e The Level of Awareness and Responsivity at 3 and 6 Months after Injury (Extended)

The level of awareness and responsivity of each patient was assessed 3 months after injury (T0) and 6 months after injury (T1). For patients older than 48 months (n = 54), this was performed through the administration of both the CNCS and LOCFAS (Supplementary Table S2). At T0, patients were diversely allocated in Levels 1, 2 and 3 of CNCS, corresponding to 'near, moderate and marked coma' for the most part. However, most of them scored into the Level 2 –'generalized response' (66.7% of the sample) of LOCFAS. At T1, almost half of the patients scored 'no coma' at the CNCS. At LOCFAS, half scored into the Level 3 –'localized response', a quarter fell into level 5 'inappropriate, not agitated behavior' and one out of five remained into Level 2 'generalized response'. The probability to fall into a certain CNCS Level resulted not to be significantly influenced by the variable "etiology" (i.e. TBI vs. NTBI), both at T0 ($\chi^2 = 5.0$, $p = 0.288$) and at T1 ($\chi^2 = 2.4$, $p = 0.498$). The same was found for LOCFAS Levels ($\chi^2 = 2.0$, $p = 0.740$ at T0; $\chi^2 = 1.1$, $p = 0.778$ at T1).

Patients younger than 48 months (n = 38) were administered the CNCS only (Supplementary Table S3). In this subgroup, CNCS scores at T0 and T1 show distributions comparable to those observed in older children. Over all the total sample of 92 children, most patients fell into the Level 2 –'moderate coma'- and the Level 1 –'near coma'- (70.7% of the total sample) of CNCS at T0. At T1, most patients fell into the Level 1 –'near Coma'- and the Level 0 –'no coma'- (80.4% of the total sample) (Supplementary Table S4). The probability to fall into certain CNCS Level still resulted not to be significantly influenced by the variable "etiology" (i.e. TBI vs. NTBI), both at T0 ($\chi^2 = 5.5$, $p = 0.242$) and at T1 ($\chi^2 = 2.0$, $p = 0.576$).

Figure 1 (main text) shows the confusion matrices (joint representation of scores at CNCS and LOCFAS) at T0 and T1, for patients aged older than 48 months. At T0, patients in CNCS Levels 2 and 3 'moderate and marked coma' were mainly scored at LOCFAS Level 2 'generalized response'; patients in CNCS Level 1 'near coma' were split between LOCFAS Levels 2 and 3 'generalized and localized response'. All cases who scored CNCS Level 0 'no coma' received LOCFAS Level 3 'localized response'. At T1, patients in CNCS Levels 2 were scored at LOCFAS Level 2; patients in CNCS Level 1 were mainly scored 3 at LOCFAS, and those in CNCS 0 scored between 3 and 5 at LOCFAS ('localized response' and above).

Supplementary Table S2. Frequencies and percent distribution of the patients' scores into the CNCS and LOCFAS levels at T0 and at T1. Results refer to patients older than 48 months (n = 54).

CNCS Level	Level of Awareness and Responsivity	T0 *n* (%)	T1 *n* (%)
**0**	**NO COMA**: the patient is consistently and readily responsive to at least 3 sensory stimulation tests plus consistently responsive to simple commands.	2 (3.7%)	25 (46.3%)
**1**	**NEAR COMA**: the patient is consistently responsive to stimulus presented through 2 sensory modalities and/or inconsistently or partially responsive to simple commands.	17 (31.5%)	21 (38.8%)
**2**	**MODERATE COMA**: the patient is inconsistently responsive to stimulation presented by 2 or 3 sensory modalities but not responsive to simple commands. He/she may vocalize (in absence of tracheotomy) with moans, groans and grunts but not with recognizable words.	19 (35.2%)	7 (13.0%)
**3**	**MARKED COMA:** the patient is inconsistently responsive to stimulation presented through 1 sensory modality and not responsive to simple commands. No vocalization.	14 (25.9%)	1 (1.9%)
**4**	**EXTREME COMA:** no responsivity to any sensory stimulation tests is present, nor response to simple commands. No vocalization.	2 (3.7%)	0 (0.0%)
**LOCFAS Level**	**Level of awareness and responsivity**	**T0 *n* (%)**	**T1 *n* (%)**
**1**	No response	3 (5.5%)	0 (0.0%)
**2**	Generalized response	36 (66.7%)	11 (20.3%)
**3**	Localized response	12 (22.2%)	27 (50.0%)
**4**	Confused-agitated behavior	1 (1.9%)	1 (1.9%)
**5**	Inappropriate, not agitated behavior	2 (3.7%)	15 (27.8%)

Supplementary Table S3. Frequencies and percent distribution of the patients' scores into the CNCS levels at T0 and at T1. Results refer to patients younger than 48 months (n = 38).

CNCS Level	Level of Awareness and Responsivity	T0 *n* (%)	T1 *n* (%)
**0**	**NO COMA**	1 (2.6%)	11 (28.9%)
**1**	**NEAR COMA**	14 (36.8%)	17 (44.8%)
**2**	**MODERATE COMA**	15 (39.5%)	7 (18.4%)
**3**	**MARKED COMA**	6 (15.8%)	3 (7.9%)
**4**	**EXTREME COMA**	2 (5.3%)	0 (0.0%)

Supplementary Table S4. Frequencies and percent distribution of the patients' scores into the CNCS levels at T0 and at T1. Results refer to the whole sample (n = 92).

CNCS Level	Level of Awareness and Responsivity	T0 *n* (%)	T1 *n* (%)
**0**	**NO COMA**	3 (3.3%)	36 (39.1%)
**1**	**NEAR COMA**	31 (33.7%)	38 (41.3%)
**2**	**MODERATE COMA**	34 (37.0%)	14 (15.2%)
**3**	**MARKED COMA**	20 (21.7%)	4 (4.4%)
**4**	**EXTREME COMA**	4 (4.3%)	0 (0.0%)

3.3e The Evolution of the State of Consciousness From 3 to 6 Months after Injury

Initially, the evolution of the state of consciousness was studied by comparing the CNCS Levels at T0 and T1 in the total sample (n = 92). Similarly, the scores for any of the 11 CNCS items were compared between T0 and T1. The average CNCS Level was 2.9 (SD = 0.9) at T0, while it decreased to 1.8 (SD = 0.8) at T1; the difference proved to be statistically significant (t = 11.0, p < 0.001), meaning that patients showed higher levels of awareness and responsivity at 6 months w.r.t. at 3 months after injury. All 11 items of CNCS significantly changed between T0 and T1 in the direction of higher awareness and responsivity, although medians remained unchanged for the *olfactory* item and *pain* was already saturated at T0. The *threat* item shifted from the lowest to the highest score; the *auditory*,

command responsivity, *visual*, *tactile* and *vocalization* items all stepped up one score (one or more units). For details see Supplementary Table S5. Analogously, in patients older than 48 months, all LOCFAS domains significantly changed between T0 and T1 in the direction of higher awareness and responsivity (see Supplementary Table S6).

Additionally, in patients older than 48 months the evolution of the state of consciousness was studied by comparing the CNCS and LOCFAS Levels at T0 and T1 (Figure 2 in the main text). According to CNCS evaluation, patients in Level 3 'marked coma' at T0 mainly moved to Levels 2 and 1 'moderate and near coma' at T1. Patients who were in Levels 2 and 1 'moderate and near coma' at T0, chiefly moved to Levels 1 and 0 'near and no coma' at T1. Considering LOCFAS, patients scoring in Level 2 'generalized response' at T0 split between Levels 2 and 3 'generalized and localized response' at T1. Patients scoring in Level 3 'localized response' at T0 split between Levels 3 and 5 'localized response and inappropriate behavior' at T1.

Supplementary Table S5. Scores for single 11 CNCS items at T0 and T1 (reported as median and mode), and results of the comparison of each item at T0 vs. T1. Results refer to the whole sample (*n* = 92).

Item	Individual parameters	Stimulus	T0		T1		Z (*p*)
			Median	Mode	Median	Mode	
1	***Auditory***	*Bell ringing 5 seconds at 10 seconds intervals.*	2	2	0	0	5.4 (<0.001)**
2	***Command Responsivity***	*Request patient to open or close eyes, mouth, or move finger, hand or leg.*	4	4	2	0	6.2 (<0.001)**
3	***Visual***	*Light flashes (1/sec x 5) in front, slightly left, right, and up and down each trial.*	4	4	2	0	6.1 (<0.001)**
4	***Visual***	*Tell patient "Look at me"; move face 20'' away, from side to side.*	4	4	1	0	5.8 (<0.001)**
5	***Threat***	*Quickly move hand forward to within 1''-3'' of eyes.*	4	4	0	0	5.5 (<0.001)**
6	***Olfactory***	*Ammonia capsule/bottle 1''under nose for about 2 seconds.*	2	4	2	0	4.5 (<0.001)**
7	***Tactile***	*Shoulder tap- tap shoulder briskly 3X without speaking to patient; each side.*	4	4	2	2	6.7 (<0.001)**
8	***Tactile***	*Nasal swab (each nostril; entrance only- do not penetrate deeply).*	1	0	0	0	4.4 (<0.001)**
9	***Pain***	*Firm pinch on finger tip; pressure of wood pencil across nail; each side.*	0	0	0	0	3.3 (0.001)*
10	***Pain***	*Robust ear pinch/pull 3X; each side.*	0	0	0	0	3.9 (<0.001)**
11	***Vocalization***	*None. Score best response.*	4	4	2	2	5.8 (<0.001)**

** highly significant at p-val<0.001. * significant at p-val<0.05.

Supplementary Table S6. Scores for the single 10 LOCFAS domains at T0 and T1 (reported as median and mode), and results of the comparison of each item at T0 vs. T1. Results refer to patients older than 48 months (n = 54).

Item	Domains	T0		T1		Z (p)
		Median	Mode	Median	Mode	
1	*Attention for environment*	1	1	2	1	4.6 (<0.001)**
2	*Responses to stimuli*	3	3	3	3	4.8 (<0.001)**
3	*Behavior*	2	2	3	3	5.1 (<0.001)**
4	*Information processing*	1	1	1	1	3.4 (0.001)*
5	*Execution of commands*	1	1	2	2	5.0 (<0.001)**
6	*Awareness of self*	3	2	3	3	5.0 (<0.001)**
7	*Time orientation*	1	1	1	1	2.6 (0.008)*
8	*Ability to perform self-care activities*	1	1	1	1	2.3 (0.023)*
9	*Ability to converse*	1	1	2	1	4.3 (<0.001)**
10	*Ability to learn new information*	1	1	1	1	3.0 (0.003)*

** highly significant at p-value < 0.001. * significant at p-value < 0.05.

3.4e The Evolution of the State of Consciousness in Patients with Stable Score at 3 and 6 Months after Injury

Of the 92 patients, 24 (26.1%) scored in the same CNCS Level at T0 and T1, thus showing no modification in their classification at the two time-points. This subgroup was further tested for possible changes of the Average CNCS Score between T0 and T1, which indeed proved to be significantly lower at T1 vs. T0 (mean at T0 = 1.8; mean at T1 = 1.5; t-value = 3.6; p = 0.002), and of the Total CNCS Score (mean at T0=20.1; mean at T1 = 16.2; t-value = 5.3; p < 0.001). Of the 24 patients who failed to show change in CNCS Level at T1, 3 remained in Level 3 'marked coma', 5 in Level 2 'moderate coma', 13 in Level 1 'near coma' and 3 patients remained in Level 0 'no coma'. Moreover, of these 24 patients, 10 where older than 48 months and had LOCFAS evaluations. Of these 10 patients, 5 showed the same LOCFAS score, and 5 showed different LOCFAS scores at T0 and T1.

Of the 54 patients assessed with both scales, 18 (33.3%) failed to show change in LOCFAS Level at T1. Of these, 10 remained in Level 2 'generalized response', 6 in Level 3 'localized response', and 2 patients remained in Level 5 'inappropriate behavior'. Of these 18 patients, 5 showed the same CNCS score at T0 and T1, and 13 showed different CNCS score at the two time-points.

3.6e The Evolution of the State of Consciousness with Respect to the Initial Ability to Follow Commands

Among the patients older than 48 months (n = 54), those classified as having 'high command responsivity' at T0 were 9 according to CNCS (score = 0 at item n°2) and 19 according to LOCFAS (score = 2, 3 of 4 at domain 'execution of commands'), with total overlap between the two sets. At T1 they were 31 according to CNCS, 45 according to LOCFAS, and total overlap.

Then, the sample was divided in two groups, according to the patients' *command responsivity* at T0. Patients who scored 'high vs. low command responsivity' according to LOCFAS at T0 were studied. We aimed to assess whether early (T0) sign of ability to follow commands is related to better scores at T1. 'High command responsivity' at T0 resulted in lower Total CNCS Scores at T0 (t = 7.8, p < 0.001) and at T1 (t = 2.9, p = 0.005), and thus better state of consciousness at both T0 and T1, with respect to the 'low command responsivity' group. *Visual* (item 3, χ^2 = 6.2, p = 0.045) and *tactile* (χ^2 = 14.2, p = 0.001; χ^2 = 9.4, p = 0.009) items of CNCS all resulted lower in the 'high command responsivity' group at T1, with respect to the 'low command responsivity' group. 'High command responsivity' at T0 was also associated to higher LOCFAS Level at T1 (t = −3.4, p = 0.001). *Responses to stimuli* (χ^2 = 11.8, p = 0.008), *Behavior* (χ^2 = 14.4, p = 0.002), *Information processing* (χ^2 = 6.8, p = 0.033), *Execution of commands* (χ^2 = 10.9, p = 0.012), *Awareness of self* (χ^2= 10.0, p=0.019), *Time orientation* (χ^2 = 11.2, p = 0.004), *Abilities to perform self-care activities* (χ^2 = 9.1, p = 0.011), *converse* (χ^2 = 9.6, p = 0.023), and *learn new information* (χ^2 = 4.9, p = 0.027) domains of LOCFAS all resulted higher in the 'high command responsivity' group at T1, indicating better state of consciousness.

3.7e The Evolution of the State of Consciousness in the Sample Divided by Etiology

The sample was divided by etiology, in order to verify whether the causes of the brain lesions had the potential to affect the evolution of the state of consciousness during the 3-months period considered in this study. Table 2 of the main text reports the scores of each CNCS item at the two assessment times (T0 and T1), both for patients with TBI and NTBI; further, the comparison of the scores at T0 vs. T1 is reported for the two groups.

The TBI group had 10 out of 11 items significantly shifted towards higher awareness and responsivity at T1 with respect to T0. The *command responsivity* item stepped up 4 scores;

The *auditory*, *visual, threat, olfactory, tactile* and *vocalization* items all gained 2 scores. Only the two items probing *pain* had unchanged medians and modes, as the descriptors already ranked top at T0; thus, no significant change between T0 and T1 was found in these cases.

The NTBI group had all items significantly improved. The *threat* item stepped up 4 scores; the *auditory, command responsivity, visual*, *tactile n*°7 and *vocalization* items all stepped up 2 scores; the *olfactory* item had improved mode and significant improvement, according to the statistical test. *Tactile n*°8 and *pain* (*n*°9 and 10) items improved, overall starting from top position at T0; however, they did not pass the strictest statistical test ($p = 0.001$).

Similarly, for patients older than 48 months, scores at LOCFAS domains in TBI and NTBI are reported in Supplementary Table S7.

Supplementary Table S7. Scores for single 10 LOCFAS domains at T0 and T1 (reported as median and mode), and results of the comparison of each item at T0 vs. T1. Results refer to samples with TBI (*n* = 37) and NTBI (*n* = 17).

		Traumatic (TBI)					Non Traumatic (NTBI)				
**Item**	**Individual parameters**	**T0**		**T1**		**Z (p)**	**T0**		**T1**		**Z (p)**
		**Median**	**Mode**	**Median**	**Mode**		**Median**	**Mode**	**Median**	**Mode**	
**1**	*Attention for environment*	1	1	2	3	4.0 (<0.001)**	1	1	2	1	2.7 (0.006)*
**2**	*Responses to stimuli*	3	3	3	3	3.6 (<0.001)**	2	2	3	3	3.5 (<0.001)**
**3**	*Behavior*	2	2	3	3	4.7 (<0.001)**	2	2	3	3	2.8 (0.005)*
**4**	*Information processing*	1	1	1	1	3.0 (0.002)*	1	1	1	1	1.6 (0.102)
**5**	*Execution of commands*	1	1	2	2	4.1 (<0.001)**	1	1	2	2	3.4 (0.001)*
**6**	*Awareness of self*	3	2	3	3	4.2 (<0.001)**	3	3	3	3	3.0 (0.003)*
**7**	*Time orientation*	1	1	1	1	2.1 (0.034)*	1	1	1	1	1.6 (0.102)
**8**	*Ability to perform self-care activities*	1	1	1	1	1.6 (0.102)	1	1	1	1	1.6 (0.102)
**9**	*Ability to converse*	1	1	2	1	3.7 (<0.001)**	1	1	2	2	2.8 (0.005)*
**10**	*Ability to learn new information*	1	1	1	1	2.4 (0.014)*	1	1	1	1	1.7 (0.083)

** highly significant at *p*-value < 0.001. * significant at *p*-value < 0.05.

3.9e Correlations between the Clinical Characteristics and the CNCS and LOCFAS Scales

After correction by "gender" and "age at injury", significantly negative correlation was found between CNCS Level at T0 and GOS-E ($\varrho = -0.221$, $p = 0.040$), and between Total CNCS Score at T0 and GOS-E ($\varrho = -0.262$, $p = 0.014$), neurosurgery ($\varrho = -0.236$, $p = 0.027$), and feeding disorders ($\varrho = -0.226$, $p = 0.035$). Positive correlation was observed between CNCS Level at T0 and tracheotomy ($\varrho = 0.237$, $p = 0.027$), and between CNCS Level at T0 and paroxysmal sympathetic hyperactivity episodes ($\varrho = 0.218$, $p = 0.042$). Total CNCS Score at T0 positively correlated with tracheotomy ($\varrho = 0.266$, $p = 0.013$), and with the occurrence of paroxysmal sympathetic hyperactivity episodes ($\varrho = 0.314$, $p = 0.003$). Positive correlations were found between LOCFAS Level at T0 and feeding disorders ($\varrho = 0.448$, $p = 0.001$), and between LOCFAS Level at T0 and GOS-E ($\varrho = 0.543$, $p < 0.001$).

At T1, significantly negative correlation was found between CNCS Level and GOS-E ($\varrho = -0.527$, $p < 0.001$), between Total CNCS Score and GOS-E ($\varrho = -0.624$, $p < 0.001$), and between LOCFAS Level and paroxysmal sympathetic hyperactivity episodes ($\varrho = -0.299$, $p = 0.037$). Positive correlations were found between LOCFAS Level and feeding disorders ($\varrho = 0.394$, $p = 0.005$), and GOS-E ($\varrho = 0.767$, $p < 0.001$).

Supplementary references

1. Teasdale, G., and Jennett, B. (1974) Assessment Of Coma And Impared Conciousness. *Lancet*, **304** (7872), 81–84.
2. Giacino, J.T., Ashwal, S., Childs, N., Cranford, R., Jennett, B., Katz, D.I., Kelly, J.P., Rosenberg, J.H., Whyte, J., Zafonte, R.D., and Zasler, N.D. (2002) The minimally conscious state: Definition and diagnostic criteria. *Neurology*, 349–353.
3. Rappaport, M., Dougherty, A.M., and Kelting, D.L. (1992) Evaluation of coma and vegetative states. *Arch. Phys. Med. Rehabil.*, **73** (7), 628–34.
4. Hagen, C., Malkmus, D., and Durham, P. (1972) The Rancho Los Amigos Levels of Cognitive Functioning. *Commun. Disord. Serv.*
5. Flannery, J. (1998) Using the levels of cognitive functioning assessment scale with patients with traumatic brain injury in an acute care setting. *Rehabil. Nurs.*, **23** (2), 88–94.
6. Jennett, B., and Bond, M. (1975) Assessment of outcome after severe brain damage. *Lancet*, **1** (7905), 480–484.
7. Pham, K., Kramer, M.E., Slomine, B.S., and Suskauer, S.J. (2014) Emergence to the Conscious State During Inpatient Rehabilitation After Traumatic Brain Injury in Children and Young Adults: A Case Series. *J Head Trauma Rehabil*, **29** (5), 44–48.
8. Patrick, P.D., Blackman, J.A., Mabry, J.L., Buck, M.L., Gurka, M.J., and Conaway, M.R. (2006) Dopamine agonist therapy in low-response children following traumatic brain injury. *J. Child Neurol.*
9. McMahon, M.A., Vargus-Adams, J.N., Michaud, L.J., and Bean, J. (2009) Effects of amantadine in children with impaired consciousness caused by acquired brain injury: A pilot study. *Am. J. Phys. Med. Rehabil.*
10. Villa, F., Colombo, K., Pastore, V., Locatelli, F., Molteni, E., Galbiati, S., Galbiati, S., and Strazzer, S. (2015) LOCFAS-assessed evolution of cognitive and behavioral functioning in a sample of pediatric patients with severe acquired brain injury in the postacute phase. *J. Child Neurol.*, **9**, 1125–34.
11. Liscio, M., Adduci, A., Galbiati, S., Poggi, G., Sacchi, D., Strazzer, S., Castelli, E., and Flannery, J. (2008) Cognitive-behavioural stimulation protocol for severely brain-damaged patients in the post-acute stage in developmental age. *Disabil. Rehabil.*
